# Interaction of Th(IV), Pu(IV) and Fe(III) with ferritin protein: how similar?

**DOI:** 10.1107/S1600577521012340

**Published:** 2022-01-01

**Authors:** Cyril Zurita, Satoru Tsushima, Pier Lorenzo Solari, Aurélie Jeanson, Gaëlle Creff, Christophe Den Auwer

**Affiliations:** a Université Côte d’Azur, CNRS, ICN, 06108 Nice, France; b Institute of Resource Ecology, Helmholtz-Zentrum Dresden-Rossendorf (HZDR), 01328 Dresden, Germany; c World Research Hub Initiative (WRHI), Institute of Innovative Research, Tokyo Institute of Technology, Meguro, Tokyo 152-8550, Japan; d Synchrotron Soleil, Saint-Aubin, F91192 Gif-sur-Yvette Cedex, France

**Keywords:** plutonium, ferritin, nuclear toxicology, X-ray absorption spectroscopy

## Abstract

Ferritin is the main protein of Fe storage in eukaryote and prokaryote cells. It is a large multifunctional, multi-subunit protein consisting of heavy H and light L subunits. In the field of nuclear toxicology, it has been suggested that some actinide elements, such as thorium and plutonium at oxidation state +IV, have a comparable ‘biochemistry’ to iron at oxidation state +III owing to their very high tendency for hydrolysis and somewhat comparable ionic radii. Therefore, the possible mechanisms of interaction of such actinide elements with the Fe storage protein is a fundamental question of bio-actinidic chemistry. We recently described the complexation of Pu(IV) and Th(IV) with horse spleen ferritin (composed mainly of L subunits). In this article, we bring another viewpoint to this question by further combining modeling with our previous EXAFS data for Pu(IV) and Th(IV). As a result, the interaction between the L subunits and both actinides appears to be non-specific but driven only by the density of the presence of Asp and Glu residues on the protein shell. The formation of an oxyhydroxide Th or Pu core has not been observed under the experimental conditions here, nor the interaction of Th or Pu with the ferric oxyhydroxide core.

## Abstract 2

The mechanisms of interaction of thorium and plutonium with Fe storage horse spleen ferritin protein (L subunit) have been described by combining modeling with EXAFS data. The interaction between the L subunits and both actinides appears to be driven by the density of the presence of Asp and Glu residues on the protein shell.

## 1. Introduction

Pierre and Marie Curie’s discovery of radium and polonium in 1898 marked the dawning of radiochemistry. From that point forward, it became a full-fledged research area, receiving international attention and playing a strategic role during the Second World War. At the heart of radiochemistry are actinides, elements that are all radioactive. These have come into particular focus since the discovery (or production) of the new element, plutonium, by Seaborg in 1941. The following year, the University of Chicago built the first nuclear reactor as part of the Manhattan Project. This effort to develop a nuclear weapon raised many questions concerning safety, the environmental impact and the effect on the biosphere as well as the human population (Morrow *et al.*, 2000 ▸). In addition to actinides, under special consideration are fission products which emit different types of particles (alpha or beta) with or without gamma radiation. But actinides have some chemical particularities with respect to their position at the bottom of the periodic table. They are, for instance, considered non-essential biological elements and at the same time bear chemical and radiological toxicity when ingested by a living organism [radiotoxicity depends on the decay scheme of the considered isotope.

Nuclear toxicological studies tend to describe the retention and excretion rates of actinides as a function of the element, its redox state and its mode of administration (injection, ingestion or inhalation) as described in the 2011 edition of *The Chemistry of the Actinide and Transactinide Elements* (Albrecht-Schmitt, 2011 ▸). Detailed studies of the biodistribution and biokinetics of actinides are indeed essential for understanding their behavior *in vivo* and for documenting their biochemical toxicity (Ansoborlo *et al.*, 2006 ▸). The main target organs of these elements have been identified, and their toxic impact assessed as a function of exposure, injection dose and the element itself. The sensitivity of various living model organisms has also been explored in mice, rats, rabbits, dogs and monkeys (Duffield & Taylor, 1986 ▸). It has been suggested that some actinides, such as thorium and plutonium at oxidation state +IV, have a biochemistry comparable to iron at oxidation state +III due to their very high tendency for hydrolysis, although this assertion is of low relevance from a purely chemical point of view. In mammals, a large proportion of Fe is associated with hemoglobin, which plays an essential role in transporting oxygen throughout the body. The remaining part is mainly found as Fe(III)–transferrin complexes (also circulating), which can cross the cell barrier by endocytosis. Once in the cell, iron is reduced under the Fe(II) form and can be used for many cellular functions and/or stored by ferritin (Taylor, 1998 ▸; May *et al.*, 1980 ▸). In the 1970s, complexation of actinides(IV) with transferrin was investigated (Stover *et al.*, 1968 ▸) and in the 1990s complexation constants were assessed (Yule, 1991 ▸). More recently, Sauge-Merle *et al.* (2017 ▸) revisited the affinity constants of Pu(IV) and Fe(III) with transferrin using an original CE-ICP-MS approach. It has been shown that transferrin can interact with Pu(IV) and form a stable complex that can cross the cell barrier (endocytosis) and thus enter the cell (Duffield & Taylor, 1986 ▸). But the specific pathways by which Pu(IV) is localized and enters the cells have never been fully understood although the ternary structure of the complex has been described (Jensen *et al.*, 2011 ▸). These mechanisms may be at the origin of actinide storage which may lead to chronic contamination.

Ferritin is the main protein of Fe storage in eukaryote and prokaryote cells (Arosio & Levi, 2002 ▸; Theil *et al.*, 2013 ▸; Andrews, 1998 ▸). It is a large multifunctional, multi-subunit protein consisting of 24 subunits composed of heavy H and light L subunits of 21 kDa and 19 kDa, respectively (Arosio *et al.*, 1978 ▸). It presents a quaternary structure of mass 474 kDa and can have different subunit compositions. For example, ferritin in the liver is composed mainly of the L subunit (about 90%). Conversely, ferritin in the heart and kidneys is composed mainly of the H subunit (Hempstead *et al.*, 1997 ▸; Arosio *et al.*, 1976 ▸). Iron–protein interaction pathways are formed by channels between subunits from the surface of the protein to its iron core. Fe(II) interacts with the protein surface (the protein ring) and then moves to the center of the protein to accumulate and form a stable core of ferric oxyhydroxide also called ferrihydrite [Fe(III)]. The maximum storage capacity is estimated in the literature at around 4500 Fe atoms in total. Previous studies have determined that up to 1800 Fe atoms may be contained in the ferric oxyhydroxide core of ferritin and the maximum capacity of the protein ring is on the order of 2800 Fe atoms (for a total of 4500) (Jutz *et al.*, 2015 ▸; Pan *et al.*, 2009 ▸).

In order to provide insight into the interaction between Pu(IV), Th(IV) and the ferritin protein at the molecular level, we combined in a previous report data from X-ray absorption spectroscopy (XAS) on the one hand and modeling on the other (Zurita *et al.*, 2021 ▸). Ferritin exhibits 8 Fe transport pores, 12 mineral nucleation sites and up to 24 oxidase sites [catalytically active (H) or inactive (L)] that produce mineral precursors from ferrous iron and oxygen. In that sense, unlike transferrin or calmodulin, for instance, it exhibits much more than a few well defined metal binding sites. We have shown recently that several complexation sites may be considered with Pu(IV) and Th(IV), leading to a stoichiometric actinide:ferritin ratio largely higher than 1 under our experimental conditions (Zurita *et al.*, 2021 ▸). Modeling of the possible complexation sites was essential because XAS data analysis only reveals average environments. Here we consider this question from another point of view by combining a more detailed analysis of the modeling than previously published with our prior XAS data for Pu(IV) and Th(IV) (Zurita *et al.*, 2021 ▸). Although Th and Pu bear similar chemistry at oxidation state +IV, the decrease of atomic radius from Th to Pu may lead to variations in the coordination environment. In light of this new data analysis, we further discuss the comparison between Fe(III), Pu(IV) and Th(IV) and address the question of how similar these three cations might be with respect to ferritin interaction. L subunits (catalytically inactive) are mainly responsible for the Fe(III) nucleation process, and this is why we have worked with horse spleen ferritin (composed mainly of L subunits). All simulations were performed with subunit L as well. Herein, the Th(IV) and Pu(IV) complexes with ferritin will be denoted F-Th and F-Pu, respectively.

## 2. Materials and methods

Horse spleen ferritin and sample preparation were described in a previous report. Its sequence is displayed in Table S1 of the supporting information. The sample described in this paper is the F–Pu complex with an F:Pu ratio equal to 1:550 and described by Zurita *et al.* (2021 ▸). This ratio ensures that saturation of the protein complexation functions has not been reached.

XAS experiments were carried out on the MARS beamline at the SOLEIL synchrotron facility which is dedicated to the study of radioactive materials. All the measurements were recorded in double-layered solution cells (200 µl) specifically designed for radioactive samples at room temperature. XAS data were acquired at the Pu *L*_III_ edge (18 057 eV). Data were processed using the *Athena* code of the *Demeter* package (version 0.9.25; Ravel & Newville, 2005 ▸). The site of Pu3(B) obtained in molecular dynamics (MD) simulation B was used as a model. EXAFS signal was fitted in *R* space between 1 Å and 6 Å without any additional filtering after Fourier transformation using a Hanning window in *k* ^2^ (2.7–10.8 Å^−1^). In all the fits, only one global amplitude factor *S*_0_^2^ and one energy threshold factor *e*_0_ were considered for all contributions. The agreement factor *R* (%) and reduced quality factor χ^2^ (*Q*) are both provided as an indication of the fit quality. Simulations of the six Pu centers obtained from simulation B were performed with the *Feff9* code using an energy threshold of 8 eV as described by Zurita *et al.* (2021 ▸).

Modeling: MD simulations were performed using the *Amber* program package (Case *et al.*, 2015 ▸) with an ff99SB force field applied to the protein. For Pu^4+^, 12–6–4 LJ-type parameters developed by Merz *et al.* were employed (Li *et al.*, 2015 ▸). For carbonate ions, additional parameters were employed (Kerisit & Liu, 2014 ▸). The protonation state of the protein was adjusted to model pH 7.4. Na^+^ ions were added to make the system electrostatically neutral. TIP3P waters were then added with a minimum water layer thickness of 10 Å. A total of 500 steps of steepest descent and 500 steps of conjugate gradient with 500 kcal mol^−1^ Å^−1^ harmonic restraint on the protein were initially conducted, after which 1000 steps of steepest descent and 1500 steps of conjugate gradient were performed without constraints. It took 40 ps heating for the system to go from 0 K to 298 K with 10 kcal mol^−1^ Å^−1^ harmonic restraint on the protein, after which another 1 ns preconditioning run was performed at 298 K without restraint on the solutes. Finally, a 150 ns MD run was performed in a periodic boundary condition in NPT ensemble at 298 K. Simulations were terminated and restarted every 5 ns to avoid artificial convergence to a particular geometry. The *SHAKE* algorithm, a 2 fs time integration step, 12 Å cutoff for non-bonded interactions and the particle mesh Ewald (PME) method were used. MD trajectory was recorded every 50 ps. Simulations were performed with two different initial structures (simulations A and B) for 150 ns MD simulation time. For both simulations, the positions of six Pu atoms relative to that of the protein reached equilibrium around halfway through the 150 ns simulation (Zurita *et al.*, 2021 ▸). Therefore, the last 75 ns MD trajectory was used for statistical sampling to ensure the equilibrated structures. The RMSD values of both simulations are given in Fig. S2 of the supporting information.

Density functional theory (DFT) calculations on Th(IV) interactions with amino acid residues as well as with carbonate ligands were performed at the B3LYP level in water through the use of the conductor-like polarizable continuum model (CPCM) using *Gaussian16* (Frisch *et al.*, 2016 ▸). The effective core potentials of Stuttgart type (small core ECP in the case of Th) and corresponding basis sets were used on all elements except hydrogen. For H, the all-electron basis set of triple-zeta quality was used.

## 3. Results

### 3.1. Molecular dynamics simulations of the ferritin–Pu complex

**Figure 1:**
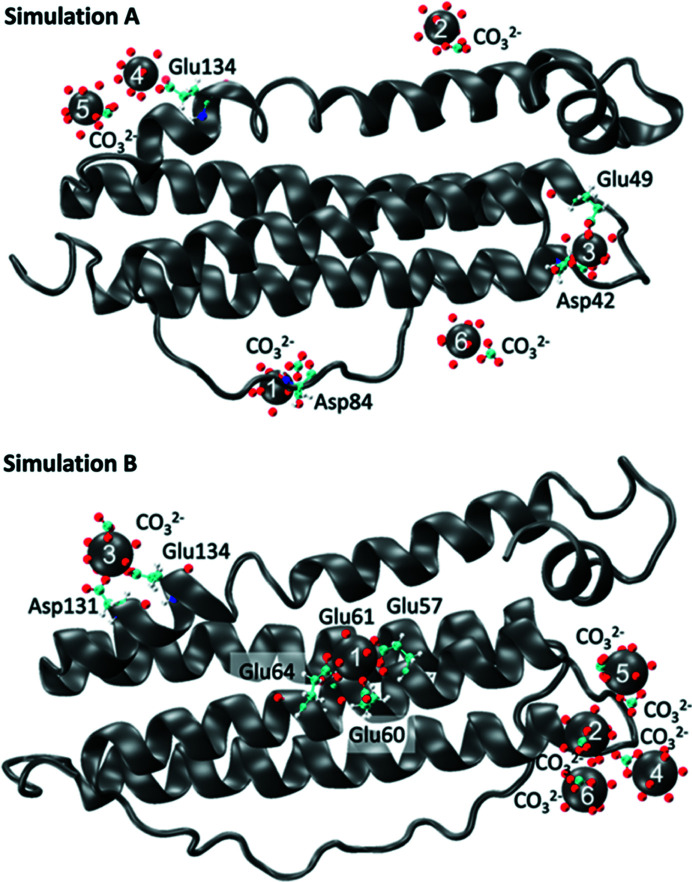
MD simulations of the F–Pu complex. Dark ribbon and dark balls depict protein and Pu atoms, respectively. Protein residues, carbonates and waters associated with Pu atoms are shown in ball-and-stick form. The hydrogen atoms of water have been omitted for clarity. The number of each Pu site refers to Figs. 2(*a*) and 2(*b*) and Table S2.

The F–Pu model was obtained from classical MD simulations and is reported by Zurita *et al.* (2021 ▸). For this model, only the L subunit has been used (L subunit of cadmium-bound ferritin, PDB entry 1aew; Hempstead *et al.*, 1997 ▸). The model is established by taking this subunit and starting from Pu^4+^(CO_3_)_5_^6−^, the anionic form in which Pu has been experimentally introduced. The results of the MD simulation of the F–Pu complexes are shown in Fig. 1 ▸. In simulation A, the L-chain structure of 1aew was used by replacing six Cd atoms initially present with Pu cations. In simulation B, six Pu cations were randomly distributed around the metal-free L-chain (see also Fig. S1). Carbonate (CO_3_^2−^) and Na^+^ ions were added to mimic the experimental carbonate concentrations and to neutralize the charges. Simulation A has been used to set the base for simulation B and allows us to observe whether the Pu atoms are stable in the predefined sites. The simulations were run for 150 ns simulation time. Simulation B stabilized slightly faster than simulation A (Fig. S2). Interestingly, the Pu(IV) interaction sites are at different positions in simulations A and B, but their coordination spheres present similarities. Pu(IV) appears to have a non-specific interaction and each Pu cation has its own environment consisting of water, carbonate and carboxyl­ate groups of the amino acids that make up the protein. In both simulations, two Pu cations interact with the protein through carboxyl­ate groups of Asp and/or Glu. The rest of the Pu (which remains unbound to the protein) forms aquo-carbonate complexes. The list of the twelve Pu sites (six for simulation A and six for simulation B) are provided in Table S2. Overall, simulation B represents the best description in agreement with the experiment *in silico*.

**Figure 2:**
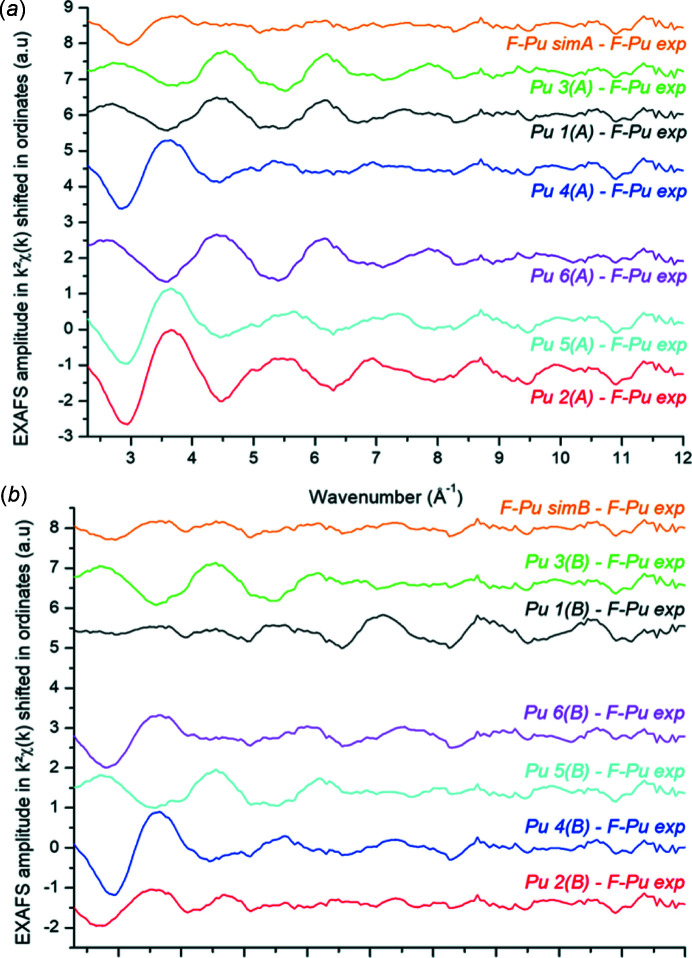
Difference EXAFS spectra χ_dif_ in *k* ^2^χ(*k*) for (*a*) simulation A and (*b*) simulation B according to the formula: χ_dif(average)_ = {F-Pu-sim(A,B) − F-Pu-exp}, χ_dif(Pu)_ = {Pu-i(A,B) − F-Pu-exp}.

The average EXAFS spectra of both simulations at the Pu *L*_III_-edge are compared in Fig. S3. Each spectrum is the average of six EXAFS spectra calculated at each Pu site (one EXAFS spectrum for each Pu atom, regardless of localization). There is no visible significant difference between both average spectra except a very small phase shift difference that will be discussed below. This strongly suggests that the average Pu environments in simulations A and B are similar but not exactly equal. Indeed, the environment of each Pu site in both simulations shows some small differences summarized in Table S2. In order to better outline the contribution of each Pu site, the calculation of the EXAFS spectra for each site for each model was performed and compared with that of the experiment, F-Pu-exp [Figs. S4(*a*) and S4(*b*)]. The complex F–Pu analyzed by EXAFS has an F/Pu ratio equal to 1/550. XAS data were acquired at the Pu *L*_III_-edge (18 057 eV) (Zurita *et al.*, 2021 ▸). More careful consideration of each spectrum at each site shows some specific differences with respect to the F-Pu-exp spectrum. Each Pu site is described in full in Table S3 and distances are provided in Table S4. Pu sites 2, 4, 5 and 6 in both simulations are different from the other Pu sites because they have no protein amino acid in their coordination sphere; those sites represent more than 50% of the Pu sites in total. They bear only ligands that are composed of water molecules and/or carbonate anions. Given the experimental conditions indicated in our previous report (Zurita *et al.*, 2021 ▸), this type of environment is unlikely to represent the majority of the Pu environments in an F–Pu complex, although it cannot be fully disregarded. On the contrary, the coordination spheres of Pu 1, 3 are composed of at least one amino acid from the protein. Furthermore, the average spectra of simulation A (F-Pu-simA) and simulation B (F-Pu-simB) appear to have a slight phase shift compared with the experimental F-Pu-exp spectrum. But this phase shift appears relatively less important for F-Pu-simB than for F-Pu-simA. This suggests that simulation B might be closer to the average Pu environment probed in the sample. To refine this trend, the F-Pu-exp experimental spectrum was subtracted one by one from each simulated spectrum, named Pu-i(A,B), and from the average spectra F-Pu-simA,B. These are displayed in Figs. 2 ▸(*a*) and 2(*b*).

The difference spectrum {F-Pu-simA − F-Pu-exp} is qualitatively more intense than {F-Pu-simB − F-Pu-exp} suggesting, as previously discussed, that simulation B better describes the average Pu–protein interaction. Also, the difference spectra of F-Pu-exp with sites Pu 1(A), Pu 3(A), Pu 1(B) and Pu 3(B) all exhibit comparable phases while difference spectra with Pu 2,4,5,6(A,B) tend to exhibit various phase shifts. Let us recall that only the Pu1 and Pu3 sites interact with the protein amino acids. This observation corroborates previous discussion that, on average, Pu in F-Pu is mainly coordinated by a mixing of carboxyl­ates (from protein amino acids), carbonates and water molecules but not by carbonates and water molecules alone. Since the F-Pu-exp spectrum clearly shows that Pu interacts with ferritin, Pu3(B) or Pu1(B) sites may be chosen as model sites, as mentioned previously. {Pu3(B) − F-Pu-exp} shows smaller amplitudes after *k* = 5 Å^−1^ than {Pu1(B) − F-Pu-exp}, justifying the choice of Pu3(B) as the best model for EXAFS data fitting, although this is only qualitative.

**Figure 3:**
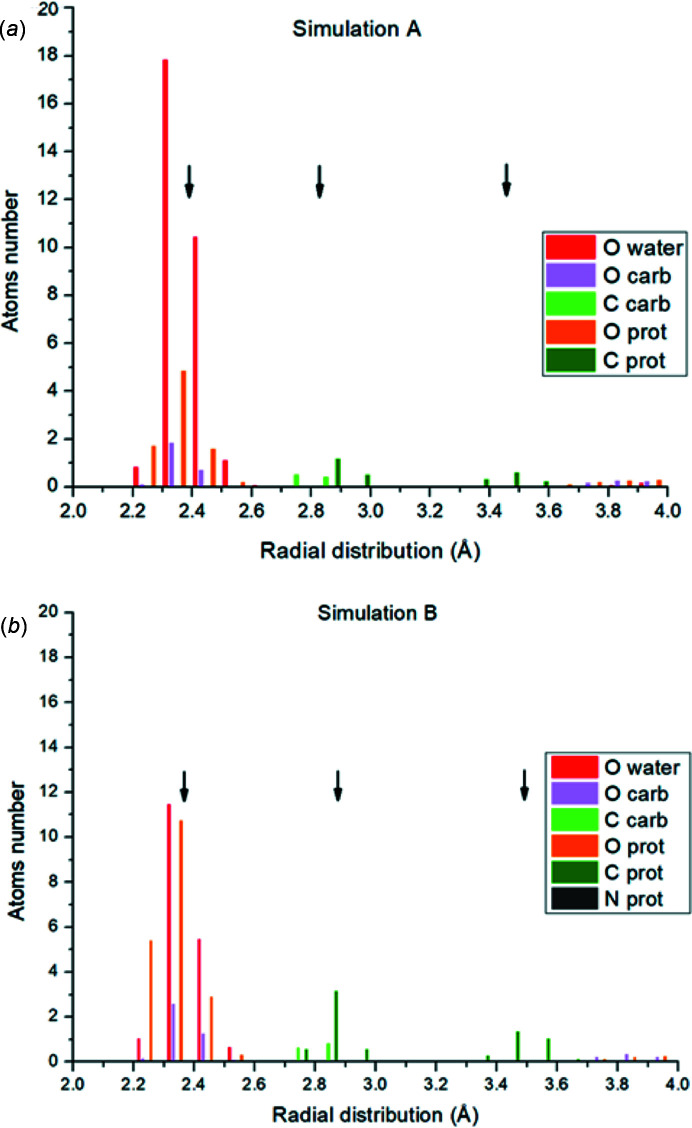
Sum of the radial distribution functions for each type of neighbour for the six Pu atoms for simulations A and B. Each vertical bar corresponds to the number of neighbors that occur at the same distance within 0.02 Å difference. The average for the first Pu—O shell and the second and third Pu—C shells are represented with a vertical black arrow (average of all Pu centers).

To further explore the coordination of Pu in simulations A and B, the distances of each type of neighbor (water, carbonate, amino acids) around each Pu site for each simulation were ranked in order to produce a sum of radial distribution functions (discrete values) for each simulation [Figs. 3 ▸(*a*) and 3(*b*)]. The radial distribution functions of simulations A and B are similar in terms of average distances (as already observed from the average simulated spectra F-Pu-simA,B) but exhibit significant differences in terms of contributors. In both simulations, the O atoms from the carboxyl­ate of the amino acid, water molecules and carbonate anions are located between 2.2 Å and 2.5 Å. But in simulation A, the number of O atoms from the water molecules largely dominates the number of O atoms from the amino acids around the Pu sites. The number of O atoms from the carbonates is comparable. The Pu⋯C shells have two distinct distances: the first between 2.7 Å and 3.0 Å and the second between 3.4 Å and 3.7 Å. The shell at the shortest distances is composed of carbons from amino acids and carbonate anions. The shell at the largest distances is only composed of carbons from amino acids. Again, in simulation B the contribution from the carbons of the amino acids is slightly dominating.

The above discussion confirms that simulation B is a better description of the interaction between Pu and the protein because it is more representative of the sample preparation, *i.e.* a random distribution of plutonium carbonate in contact with apo-ferritin (with no saturation of the protein complexation functions). But this conclusion at this point is only qualitative, keeping in mind that EXAFS sees averaged configurations and/or an average of complexes. EXAFS data fitting of F-Pu-exp was performed [with the model of Pu 3(B)] as described in our previous report and briefly above. This Pu site interacts with the different environments: water, amino acids and carbonate (Zurita *et al.*, 2021 ▸). From the fit of the EXAFS data, the total coordination number (CN) is difficult to determine with accuracy better than *ca* 20% because An(IV) CNs are large, flexible and can range from 8 to 12. We chose to fix the value to an average of 10. Best fit data already shown in our previous report give an average Pu—O distance equal to 2.39 (5) Å (Zurita *et al.*, 2021 ▸). In both simulations and as shown in Figs. 3 ▸(*a*) and 3(*b*) by arrows, the Pu—O average distance is 2.39 Å for simulation A and 2.38 Å for simulation B (average of all the Pu—O distances in the simulation). Both values agree very well with the fitted Pu—O distance. The second coordination sphere reported gives 5.2 (20) C atoms located at 3.34 (4) Å (Zurita *et al.*, 2021 ▸). Note that the uncertainty is very large as discussed in the literature (Zurita *et al.*, 2021 ▸). In the simulations, two distinct Pu—C distances are observed (average of two distinct Pu—C distributions in the simulation). The first carbon layer has an average distance of 2.82 Å for simulation A and 2.83 Å for simulation B. This layer appears to be associated with carbons of carbonates as well as carbons of bidentate amino acids. The second carbon layer in the simulations has an average distance of 3.44 Å for simulation A and 3.51 Å for simulation B. This distance corresponds to carbons of amino acids bound in a monodentate pattern and is related to the second layer of carbon observed in the distribution functions. Although the value obtained from the EXAFS fit lies 0.1 Å shorter than the second carbon layer in the simulations, this is a reasonably fair agreement. The flexibility of the protein chain and therefore the variations in amino acid position within the Pu coordination sphere may explain this difference.

### 3.2. Quantum chemistry of Th–amino acid complexes

To further explore possible differences between the behavior of Pu and Th, *ab initio* quantum chemical calculations (DFT) of the complexes of Th with the amino acids involved in the ferritin ring (Asp and Glu mostly) were performed (Table S5). Only complexes that also include at least one water molecule have been taken into account. The complex Th(CO_3_)_4_(H_2_O)_2_^4−^ was also simulated for comparison. Pu^4+^ has partially filled 5*f* orbitals and is difficult to treat with DFT, whereas Th^4+^ takes a formal 5*f* ^0^ electronic configuration and can be handled easily with DFT. Therefore, the idea here is to complement MD work on F-Pu with DFT work on F-Th. Table S5 shows the coordination sphere of each Th complex and Table S6 summarizes the distances obtained in those complexes with Asp and Glu amino acids. The average Th—O distances in all complexes are very similar, whatever the composition of the complex. But differences appear regarding the composition of each complex (amino acid, carbonate and water). The same is true for the Th—C distances. For instance, the Th—O distance of the amino acids varies according to the type of binding: monodentate *versus* bidentate interaction. As expected, the monodentate interaction has a shorter distance (*ca* 2.33 Å) than the bidentate (*ca* 2.62 Å). In contrast, the mode of interaction of the amino acids seems to have little influence on the Th—O distance of the water molecules. Similarly, the Th—C distance for the amino acid monodentate interaction is larger (3.56 Å) than for the bidentate interaction (3.03 Å). Interestingly, the Th—C distance in Th(CO_3_)_4_(H_2_O)_2_^4−^ is significantly lower (more than 0.2 Å) than in all other complexes involving amino acids.

EXAFS data fitting of F-Th-exp was performed in the same way as for F-Pu-exp and is shown in our previous report (Zurita *et al.*, 2021 ▸). The first layer is composed of ten oxygens (fixed value) at 2.44 Å. This average distance is slightly shorter (about 0.1 Å) than that obtained from the DFT models of Th with different environments (Table S6). This could be for several reasons: (i) the average distance calculated for the selected complexes is obviously not fully representative of the protein structure, (ii) solvent effects and protein conformation are not taken into account. The second sphere is composed of 5.1 (22) C at an average distance of 3.56 Å. This value corresponds to Th—C distances closer to a monodentate inter­action than to a bidentate interaction. Of note is the great uncertainty in this distance associated with the EXAFS data. For the carbonate complex, the Th—C EXAFS distance obtained for the limit complex Th(CO_3_)_5_^6−^ is, as expected, significantly larger than the Th—C distance in the ternary model. In summary, EXAFS data compared with the DFT models suggest that the interaction modes of amino acids with Th cannot be discriminated. Indeed, the Th—C distance of 3.56 Å has a high uncertainty which does not exclude, as in the EXAFS F-Pu data and the associated MD calculation, a mixture of amino acid interaction modes.

## 4. Perspective on the environment of Fe in ferritin: comparison with Pu and Th

Iron in ferritin has been observed and described for decades, particularly how amino acids from the surface of the protein interact with Fe cations in the ferroxidase center in order to form the ferric oxyhydroxide core. The ferroxidase center consists of two interaction sites A and B (similar but slightly different environment). Fe(II) in site A interacts with a monodentate glutamate residue, a histidine residue, a water molecule and a bridging glutamate residue between sites A and B (Le Brun *et al.*, 2010 ▸). Fe(II) in site B interacts with two monodentate glutamate residues and the bridging glutamate residues. An oxo- or hydroxo-bridge occurs between sites A and B (Le Brun *et al.*, 2010 ▸). But a third iron-binding site, C (Stillman *et al.*, 2001 ▸), includes an Fe polyhedron composed of four glutamate residues and two water molecules (Le Brun *et al.*, 2010 ▸). Finally the ferroxidase center acts as a gated iron pore after oxidation of Fe(II) by transferring the resulting Fe(III) into the cavity.

In contrast to this description, the L subunits do not oxidize Fe(II) and they exhibit a cluster of four glutamate residues on their inner surface (Chasteen & Harrison, 1999 ▸). The Fe_A_—Fe_B_ inter-iron distance is on the order of 3.24 Å (3.18 Å to 3.27 Å) (Crow *et al.*, 2009 ▸) and the Fe_B_—Fe_C_ inter-iron distance is on order of 5.79 Å (5.73 Å to 5.84 Å) (Stillman *et al.*, 2001 ▸), though the occupancy of Fe_B_ is low relative to Fe_C_. Note that the inter-iron distance varies slightly according to the nature of the ferritin (bacterioferritin) and their origin (spleen, heart, liver) but also the species studied (horse, human, frog). Site C has an important effect on the characteristics of iron mineralization (Treffry *et al.*, 1998 ▸). To the best of our knowledge, there is no example of structural analysis of an Fe coordination sphere within sites A, B or C. But from the above description it is clear that carboxyl­ate functions play an essential role in iron binding in both sub­units.

Other structural models of human H subunit and frog mutated H subunit containing divalent cations such as Zn(II), Co(II), Cu(II), Mn(II) or Mg(II) have been studied. In short, the divalent metal cations could be used for inhibiting the ferroxidase activity within the H subunits (Tosha *et al.*, 2010 ▸; Behera & Theil, 2014 ▸). Structural investigations have shown that the cation environments are similar to those of iron but with significantly larger inter-cation distances (Liu & Theil, 2005 ▸).

**Figure 4:**
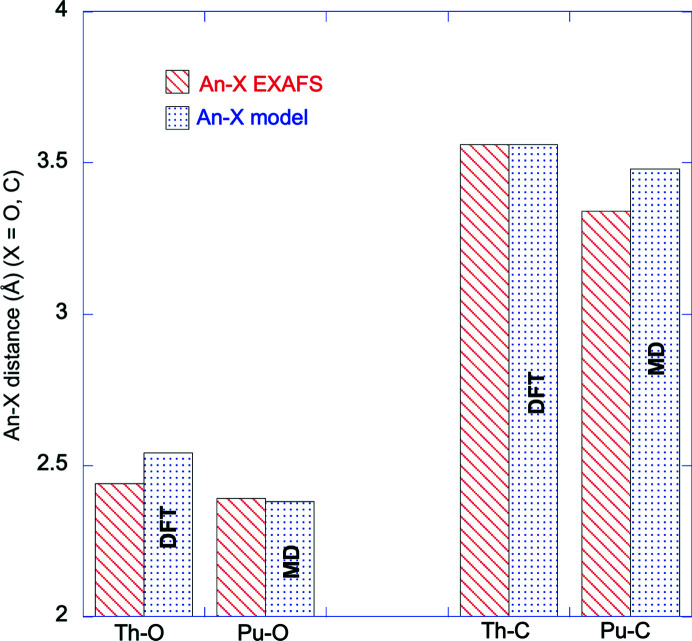
Comparison of the various distances obtained for the F-Th and F-Pu complexes. Data obtained with EXAFS data fitting (Zurita *et al.*, 2021 ▸) and data obtained from simulation (MD for Pu and DFT for Th).

To the best of our knowledge, structural data of exogeneous cations in interaction with L subunits are lacking. With the above combination of modeling and EXAFS data analysis, we bring a new perspective on the Th/Pu interaction with ferritin compared with our previous paper on the subject (Zurita *et al.* 2021 ▸). The L subunit selected for this work does not have a ferroxidase center but has similar interaction sites as mentioned above (Chasteen & Harrison, 1999 ▸). In both of our simulations A and B with plutonium, Pu2 and Pu5 on the one hand and Pu4 and Pu6 on the other are 6.0 Å and 6.3 Å apart, respectively. The significant Pu–Pu distance may suggest that these atoms behave the same way as Fe(III) when interacting at the surface of the protein ring and with ferroxidase. Also, none of the EXAFS spectra of F-Pu and F-Th complexes suggest the presence of An–An interaction (this would mean distances less than 4–5 Å for EXAFS). But under our experimental conditions, there is no evidence for the formation of a Pu, nor a Th oxyhydroxide core (the possible interaction of Pu or Th with the ferric oxyhydroxide core is discussed further below). A summary of the average distances involved in Th and Pu coordination spheres in F-Th and F-Pu complexes (experiment and simulation) is represented in Fig. 4 ▸ [for Pu: simulation B, average of all Pu sites, 〈Pu—O〉 = 2.38 Å and 〈Pu—C〉 = 3.51 Å as in Fig. 3 ▸(*b*); for Th: average distances from complexes Th2 and Th4 of Table S5, 〈Th—O〉 = 2.53 Å and 〈Th—C〉 = 3.56 Å]. For both O and C shells, the systematic decrease of distances from Th to Pu is in agreement with (and of the order of) actinide contraction. Overall, the agreement between EXAFS data and models (MD for Pu and DFT for Th) is very satisfactory and discrepancies are within 0.1 Å (for Th—O and Pu—C). One should keep in mind in this comparison that, in all models, at least one water molecule is included and therefore the average An—O distance results from an average between the water and protein contributions. The presence of a carbonate anion within the An coordination sphere is possible but is difficult to assess according to this dataset.

One may propose at this point that Pu(IV), Th(IV) and Fe(II) interact with the protein ring *via* the carboxyl­ate functions of mainly Asp and Glu amino acids. Fig. S1 of the L subunit shows that the Pu atoms are either directly interacting with at least one Asp and one Glu amino acid (or both) or gravitating close to those residues. Conservation of the glutamate and aspartate residues in both subunits (Chasteen & Harrison, 1999 ▸) underline their potential role in cation binding. Apart from the Pu1B site which interacts with three Glu, the other Pu centers are positioned rather close to the edge of the subunit. Due to their position, these cations could also interact with amino acids from other subunits. However, as there is no reported evidence of Pu/Th interaction with ferritin *in vitro*, it is impossible to conclude here on further mechanisms regarding the H subunit, nor to fully describe the mechanisms of interaction of both actinides with the triple channel.

In our previous report (Zurita *et al.*, 2021 ▸) XANES spectra at the Fe *K*-edge were performed for native F, F-Pu and F-Th complexes (F-Pu has not been measured, for technical reasons) in order to monitor possible changes in the iron core of the protein after Th complexation. No difference has been shown from this spectrum between native F and F-Th. A clear change in the XANES spectrum at the Fe *K*-edge would imply that most Fe environments would have been affected by the presence of Th atoms, and this is not the case. Under our experimental conditions (Th/Fe ratio = 0.5), this means that most Fe atoms are located a significant distance from the Th complexation sites (far meaning more than 4–5 Å from the XAS point of view). The similarity between Th and Pu data described throughout the paper suggests that the same conclusion can be drawn for plutonium as well.

To conclude, it can be assumed that Th/Pu interact with mainly Asp and Glu amino acids of the protein ring (here, L subunits) in a similar way that iron does. But in the case of the entire protein, this interaction might not be specific and only driven by the density of Asp and Glu residues on the protein shell. The formation of an oxyhydroxide Th or Pu core was not observed under our experimental conditions, nor the interaction of Th or Pu with the ferric oxyhydroxide core. This demonstrates that, uder our conditions, Th or Pu are not driven through the triple channel towards the inorganic core of the protein. Considering the effective ionic radii for Th^4+^ and Pu^4+^ (CN = 8) indicates a decrease from 1.05 Å (Th) to 0.96 Å (Pu) that is reflected by the small decrease in the average distances of Fig. 4 ▸. In contrast, Fe^3+^ (CN = 6) has a smaller radius (0.645 Å) although with a smaller CN (Shannon, 1976 ▸). It should be kept in mind that our reasoning is based on the modeling of a single subunit and not on a whole ferritin. We may therefore hypothesize that the flexibility of the glutamate residues of the L subunits can accommodate this difference in ionic size. Finally, the similarity between Th and Pu data (*modulo* actinidic contraction) suggests that both cations behave the same way towards the L subunit of ferritin.

Back to the title question of this report, considering iron homeostasis as a guide for plutonium biochemistry is certainly too adventurous. But similarities appear in their affinity for aspartate and glutamate residues when enough flexibility can accommodate the difference in polyhedron shape and size between both cations.

## Supplementary Material

Additional figures and spectra (Figs. S1 to S4; Tables S1 to S6). DOI: 10.1107/S1600577521012340/ok5058sup1.pdf
