# Supplementary material for: Interaction of Th(IV), Pu(IV) and Fe(III) with ferritin protein: how similar?
Source: J Synchrotron Radiat. 2022 Jan 1;29(Pt 1):45–52. doi: 10.1107/S1600577521012340 (PMC8733997; doi:10.1107/S1600577521012340)
Supplement: Supplementary file 1 [file s-29-00045-sup1.pdf]

JOURNAL OF SYNCHROTRON RADIATION

Volume 29 (2022)

Supporting information for article:

Interaction of Th(IV), Pu(IV) and Fe(III) with ferritin protein: how similar?

Cyril Zurita, Satoru Tsushima, Pier Lorenzo Solari, Aurélie Jeanson, Gaëlle Creff and Christophe Den Auwer

Table S1 : Ferritin sequence (horse spleen ferritin) (Hempstead *et al.*, 1997)

Ferritin sequence (horse spleen ferritin) from PDB reference 1aew (Hempstead *et al.*, 1997). Ferritin is composed of 24 subunits from two different types: heavy (H) and light (L). In the liver and in the spleen the L subunit dominates (up to 90%). In horse spleen, the repartition is as follows: 20-22 L subunits, 2-4 H subunits.

H subunit:
Length:182
Mass (Da):21,269

TTAFPSQVRQNYHQDSEAAINRQINLELHASYVYLSMSFYFDRDDVALKNFAKYFLHQSHEE
REHAEK
LMKLQNQRGGRIFLQDIKKPDQDDWENGLKAMECALHLEKNVNESLLELHKLATDKNDPHL
CDFLET HYLNEQVKAIKELGDHVTNLRRMGAPESGMAEYLFDKHTLGECDES

L subunit
Length:175
Mass (Da):19,978

SSQIRQNYST**E**V**E**AAVNRLVNLYLRASYTYLSLGFYF**D**R**DD**VAL**E**GVCHFFR**E**LA**EE**KR**E**GA
ERLLKM
QNQRGGRALFQ**D**LQKPSQ**DE**WGTTL**D**AMKAAIVL**E**KSLNQALL**D**LHALGSAQA**D**PHLC**D**FL
ESHFL**D** **EE**VKLIKKMG**D**HLTNIQRLVGSQAGLG**E**YLF**E**RLTLKH**D**

M : Methionine	H : Histidine	I : Isoleucine	Y : Tyrosine
S : Serine	K : Lysine	R : Arginine	L : Leucine
Q : Glutamine	**E : Glutamic acid**	N : Aspargine	**D : Aspartic acid**
P : Proline	T : Threonine	C : Cysteine	F : Phenylalanine

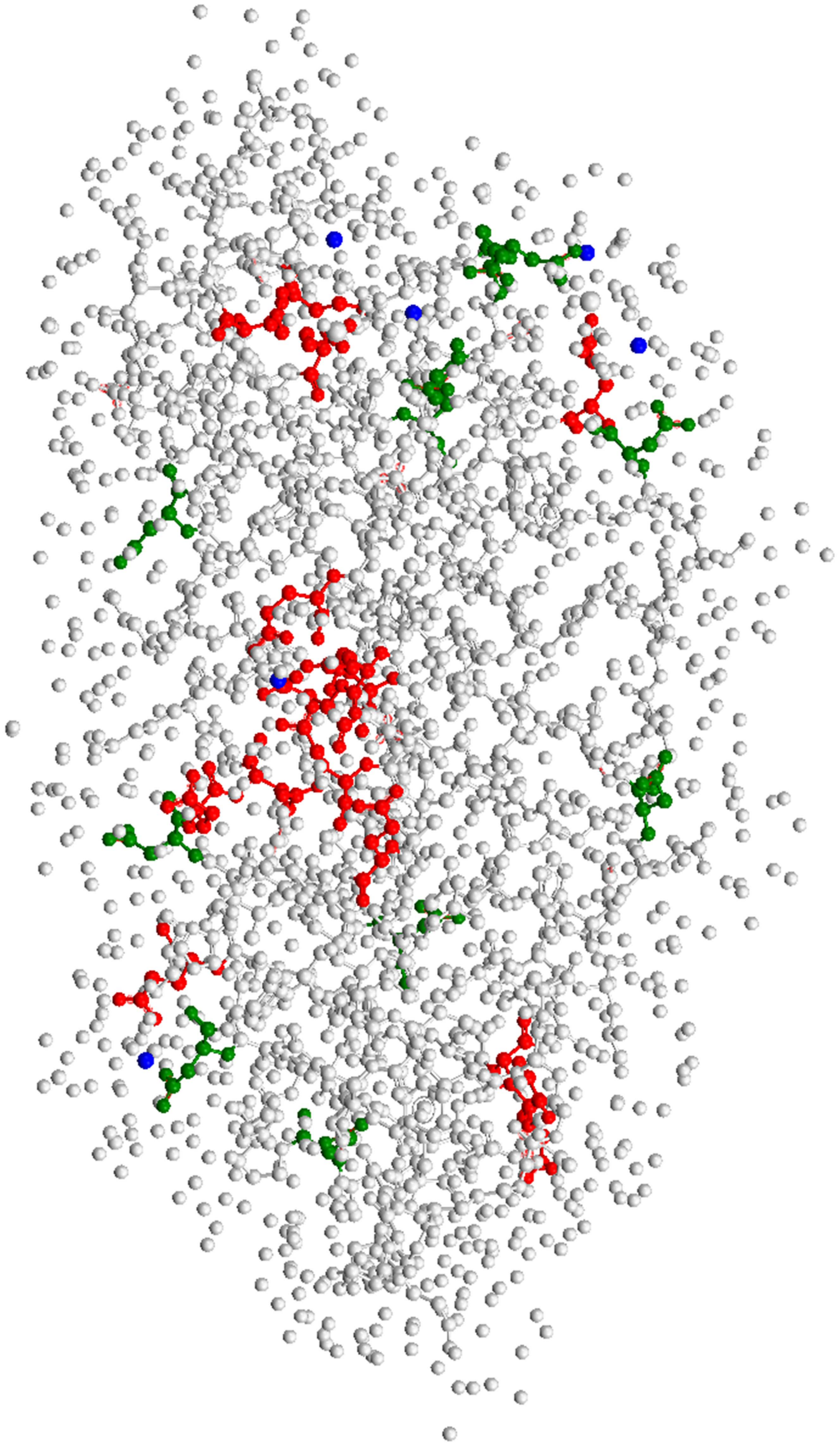

Figure S1 : Molecular dynamics (MD) simulation B of F-Pu complex, L subunit. Some of the amino acid residues are highlighted : Glu in red, Asp in green. Pu atoms are in blue.

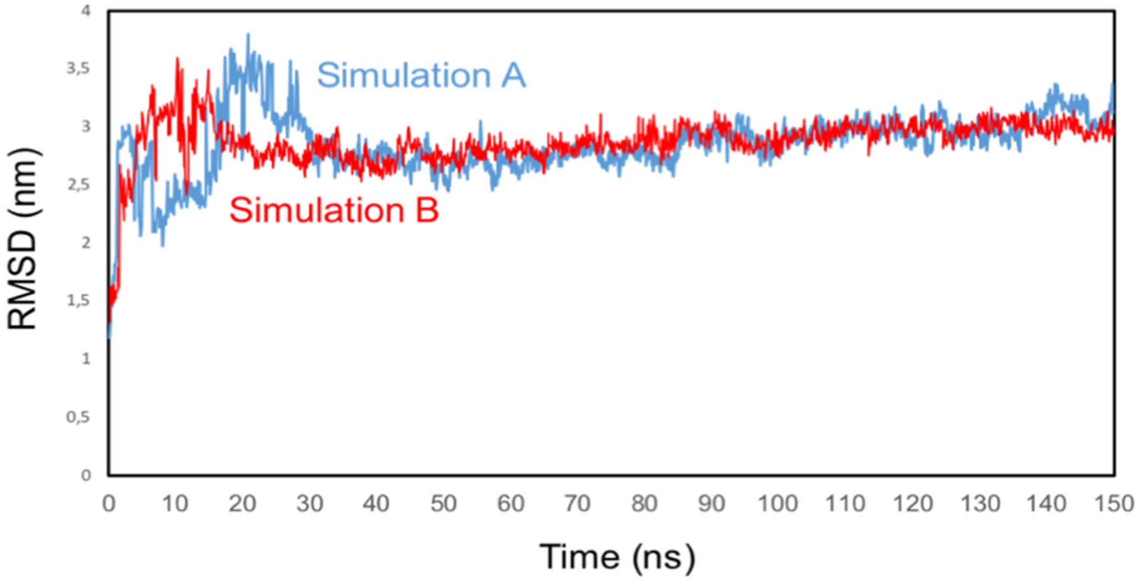

Figure S2 : Time evolution of Root Mean Square Deviation (RMSD) (excluding waters, carbonate, and sodium ions) of Molecular dynamics (MD) simulations A and B.

Table S2 : Local environment (type of ligand and amino acid from the protein) of each Pu site of MD simulations A and B. Numbers refer to Figure 1.

Pu site	Simulation A	Simulation B
**Pu 1**	Glu, water	Glu, water
**Pu 2**	Carbonate, water	Carbonate, water
**Pu 3**	Asp, Glu, water	Asp, Glu, Carbonate, water
**Pu 4**	Carbonate, water	Carbonate, water
**Pu 5**	Carbonate, water	Carbonate, water
**Pu 6**	Carbonate, water	Carbonate, water

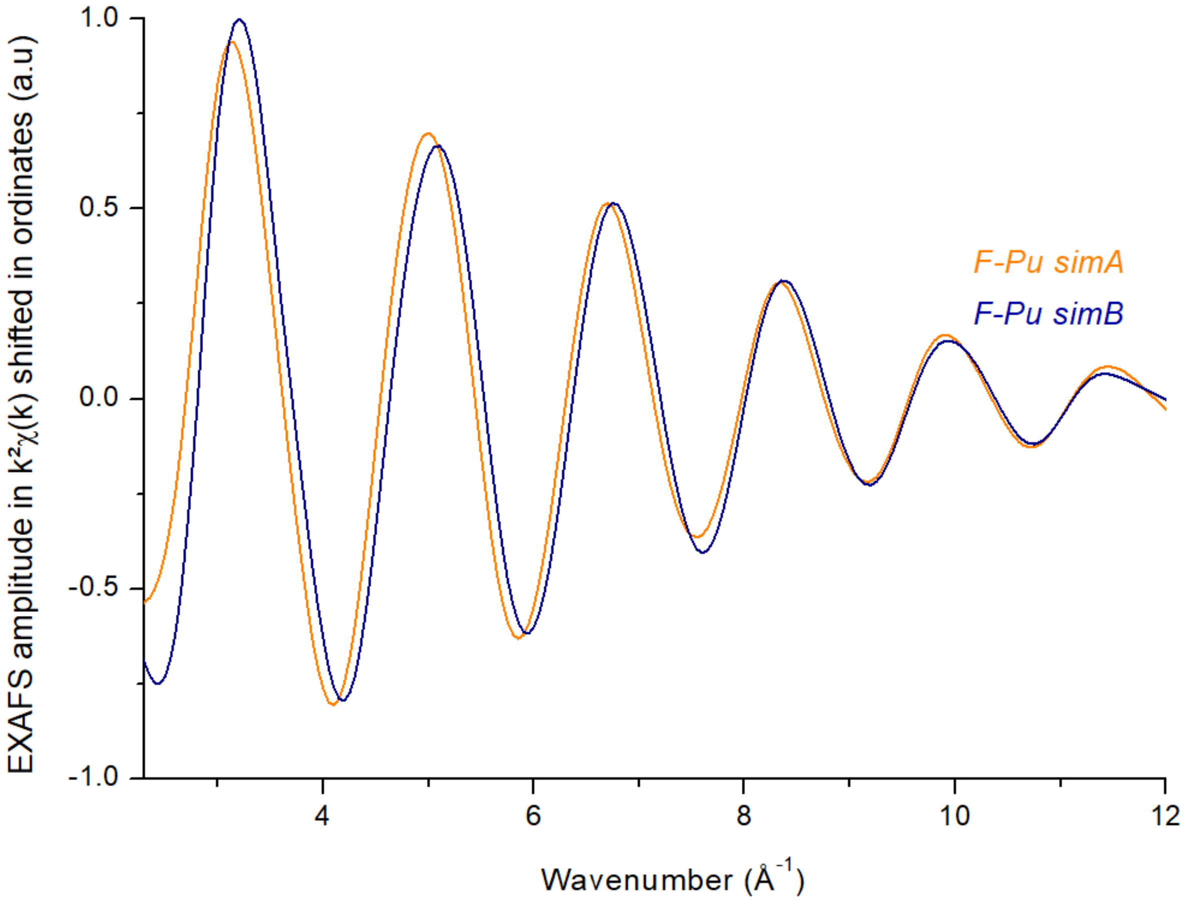

Figure S3 : Calculated EXAFS spectra in $k^2\chi(k)$ of *F-Pu simA* (orange line) and *F-Pu simB* (blue line).

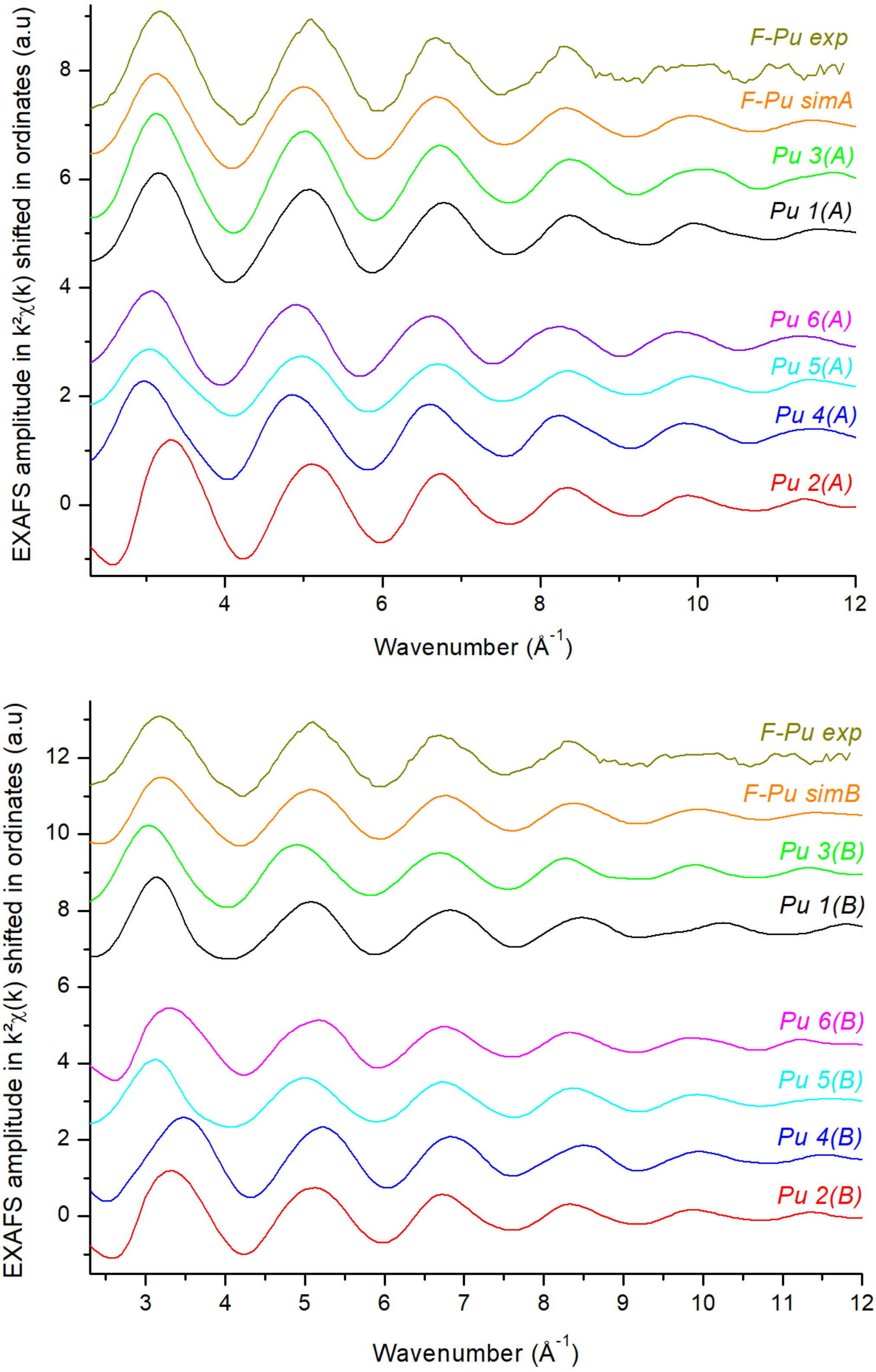

Figure S4ab : Calculated EXAFS spectra in $k^2\chi(k)$ of the 6 Pu sites contained in F-Pu simA (top) and F-Pu simB (bottom). Comparison with the average calculation of Figure SI3 and experimental spectra.

Table S3 : Structure and local interaction of each plutonium in both simulations (F-Pu simA and F-Pu simB) calculated by MD simulation. Plutonium is in blue balls, carbon in grey balls, oxygen in red balls and nitrogen in purple balls in all pictures.

	**Simulation A**	**Simulation B**
**Pu 1**<br>**A** : 3 Glu (7 O) + 3 Water (3 O)<br>**B :** 3 Glu (7 O) + 3 Water (3 O)	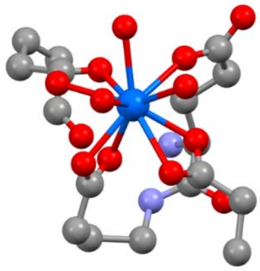	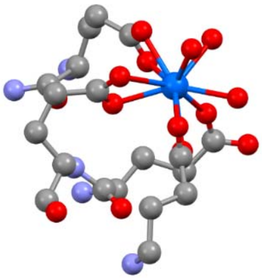
**Pu 2**<br>**A :** 1 carb (2 O)<br>+ 8 Water (8 O)<br>**B :** Pu 2(B) : 1 carb (2 O)<br>+ 8 Water (8 O)	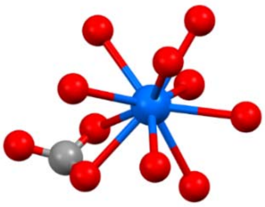	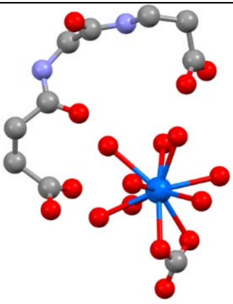
**Pu 3**<br>**A** : 1 Asp (1 O) + 1 Glu (2 O) +<br>7 Water (7 O)<br>**B** : 1 Asp (1 O) + 1 Glu (1 O) +<br>1 carb (2O) + 6 Water (6 O)	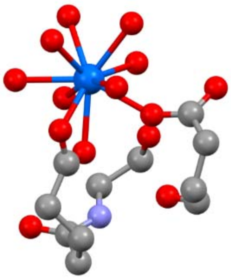	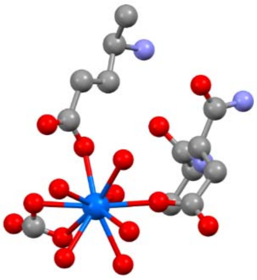
**Pu 4**<br>**A** : 1 carb (2 O) + 7 Water (7 O)<br>**B** : 1 carb (2 O) + 7 Water (7 O)	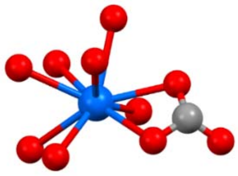	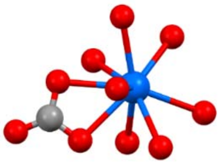
**Pu 5**<br>**A :** 2 carb (4 O) + 3 Water (3 O)<br>**B** : 2 carb (4 O) + 4 Water (4 O)	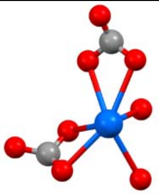	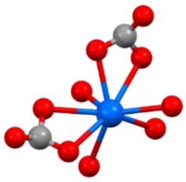
**Pu 6**<br>**A** : 1 carb (2 O) + 7 Water (7 O)<br>**B** : 1 carb (2 O) + 6 Water (6 O)	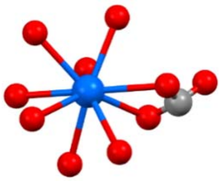	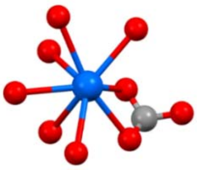

Table S4 : Summary of the distances obtained with MD calculations of F-Pu for simulation B.

	First shell Pu-O distance (Å)				Second shell Pu-C distance (Å)		
	Pu-O water	*Pu-O amino acid Mono / bidentate*	*Pu-O carb*	***Pu-O average***	*Pu-C amino acid Mono / bidentate*	*Pu-C carb*	***Pu-C average***
**EXAFS**							
F-Pu*				**2.39(5) Å**			**3.34(4) Å**
Pu carb*				**2.44(2) Å**			**2.88(1) Å**
**Model**							
Pu 1(B)	2.345 Å	2.339 / 2.380 Å	-	**2.365 Å**	3.502 / 2.872 Å	-	**3.03 Å**
Pu 2(B)	2.423 Å	-	2.339 Å	**2.406 Å**	-	2.739 Å	**2.739 Å**
Pu 3(B)	2.471 Å	2.303	2.386 Å	**2.385 Å**	3.402	2.828 Å	**3.211 Å**
Pu 4(B)	2.389 Å	-	2.406 Å	**2.392 Å**	-	2.851 Å	**2.851 Å**
Pu 5(B)	2.376 Å	-	2.358 Å	**2.367 Å**	-	2.793 Å	**2.793 Å**
Pu 6(B)	2.407 Å	-	2.421 Å	**2.411 Å**	-	2.790 Å	**2.790 Å**

*data published (Zurita *et al.*, 2021).

Table S5 : Structure and local interaction of each thorium complex calculated by DFT. Thorium is in blue balls, carbon in grey balls, oxygen in red balls and nitrogen in purple balls in all pictures.

**Th 2**<br>1 monodente Asp (1 O)<br>+ 8 Water (8 O)	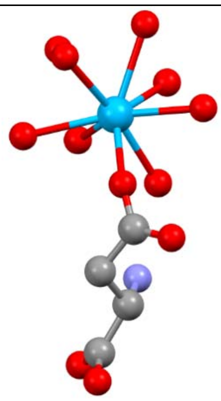	**Th 1**<br>1 bidente Asp (2 O)<br>+ 8 Water (8 O)	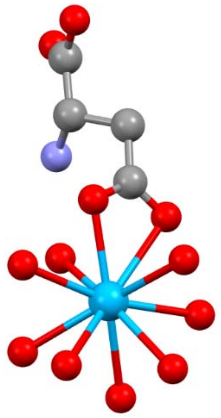
**Th 4**<br>1 monodente Glu (1 O)<br>+ 9 Water (9 O)	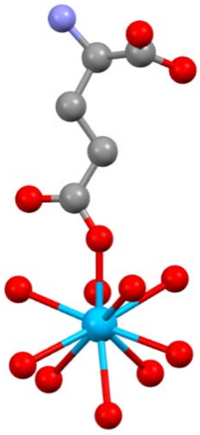	**Th 3**<br>1 bidente Glu (2 O)<br>+ 8 Water (8 O)	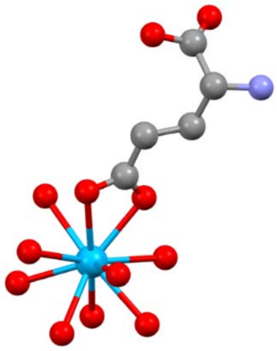
**Th-carb**<br>4 carbonates (8 O)<br>+ 2 Water (2 O)	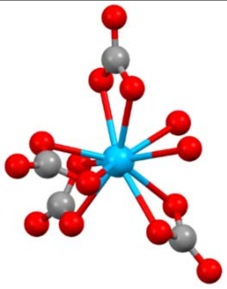		

Table S6 : Summary of the distances obtained with DFT calculations of Th with Asp and Glu amino acids and carbonate anions that also include water molecules.

	**First shell Th-O distance (Å)**				**Second shell Th-C distance (Å)**		
	Th-O water	*Th-O amino acid*	*Th-O carb*	***Th-O average***	*Th-C amino acid*	*Th-C carb*	***Pu-C average***
**EXAFS**							
F-Th exp*				**2. 44(6) Å**			**3.56(26) Å**
Th carb*				**2.50(8) Å**			**2.96(9) Å**
**Model**							
Th 1	2.56 Å	2.62 Å	-	**2.57 Å**	3.03 Å	-	**3.03 Å**
Th 2	2.55 Å	2.28 Å	-	**2.52 Å**	3.56 Å	-	**3.56 Å**
Th 3	2.54 Å	2.61 Å	-	**2.56 Å**	3.06 Å	-	**3.06 Å**
Th 4	2.57 Å	2.38 Å	-	**2.56 Å**	3.56 Å	-	**3.56 Å**
Th-carb	2.52 Å	-	2.63 Å	**2.54 Å**	-	2.77 Å	**2.77 Å**

*data published (Zurita *et al.*, 2021).
